# Perioperative Complications in Infective Endocarditis

**DOI:** 10.3390/jcm12175762

**Published:** 2023-09-04

**Authors:** Henning Hermanns, Tim Alberts, Benedikt Preckel, Magnus Strypet, Susanne Eberl

**Affiliations:** Department of Anesthesiology, Amsterdam UMC, Location AMC, Meibergdreef 9, 1105 AZ Amsterdam, The Netherlands; h.hermanns@amsterdamumc.nl (H.H.); b.preckel@amsterdamumc.nl (B.P.); m.strypet@amsterdamumc.nl (M.S.); s.eberl@amsterdamumc.nl (S.E.)

**Keywords:** infective endocarditis, complications, cardiac surgery, anesthesia, perioperative care

## Abstract

Infective endocarditis is a challenging condition to manage, requiring collaboration among various medical professionals. Interdisciplinary teamwork within endocarditis teams is essential. About half of the patients diagnosed with the disease will ultimately have to undergo cardiac surgery. As a result, it is vital for all healthcare providers involved in the perioperative period to have a comprehensive understanding of the unique features of infective endocarditis, including clinical presentation, echocardiographic signs, coagulopathy, bleeding control, and treatment of possible organ dysfunction. This narrative review provides a summary of the current knowledge on the incidence of complications and their management in the perioperative period in patients with infective endocarditis.

## 1. Introduction

Infective endocarditis (IE) is a mostly bacterial infection that affects the native heart valves, endocardial surface, or prosthetic valves. Although it is a rare disease, its incidence is increasing, and mortality rates remain high despite advances in medical treatment [1]. Early diagnosis based on physical examination, imaging, and microbiologic studies is essential to initiate successful treatment. Antibiotics are the cornerstone of the treatment of IE, with additional surgery being necessary for approximately half of the patients. Especially in the perioperative period, serious IE-related complications occur frequently and may have a significant impact on outcome [2]. However, the literature focusing on the perioperative care of patients undergoing surgery for IE is limited [3,4].

The mortality rate for patients with IE remains high, ranging from 10% to 20% in-hospital and up to 30% at one-year follow-up [1]. Multiple factors have a significant influence on increased mortality, including, among others, *S. aureus* infection, the abundance of comorbidities, older age, neurologic or pulmonary complications, embolic events, and renal dysfunction [5,6].

**Figure 1:**
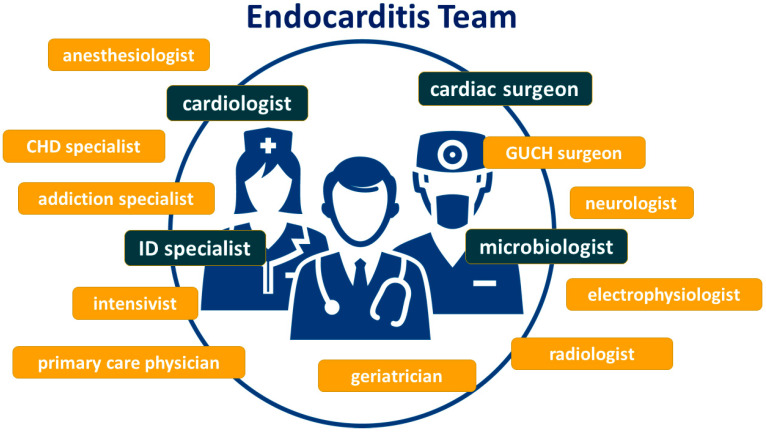
Specialists most commonly involved in multidisciplinary endocarditis teams. Highlighted in blue, medical specialists who are usually considered to be the core endocarditis team; highlighted in yellow, medical specialists who are additionally consulted in selected cases [2,10]; CHD, congenital heart disease; GUCH, grown-up congenital heart; ID, infectious disease.

The complex nature of the disease, the high morbidity and mortality, and the multitude of specialists that are commonly involved in treating these patients implies that the management of patients with IE requires a multidisciplinary approach involving cardiologists, infectious disease specialists, microbiologists, cardiac surgeons, and anesthesiologists, among others (Figure 1). Endocarditis teams warrant the multidisciplinary collaboration of healthcare professionals who are specialized in the diagnosis, treatment, and management of IE. The implementation of these teams has been shown to improve outcomes for patients suffering from IE [7,8,9] and is strongly recommended [2].

Cardiac surgery is indicated in about half of patients with IE. The indications for surgery are acute heart failure (due to valve disease), uncontrolled infection, and prevention of systemic embolic events [2,11].

The indication and timing of surgery should be carefully considered, as early surgery may be necessary in patients with rapidly progressive infections or severe valve dysfunction. However, surgery may need to be delayed in patients who require further medical management or stabilization of their condition. The perioperative period in IE is frequently associated with a multitude of complications, associated with patient-, pathogen-, and procedure-related risk factors [3].

The aim of the present narrative review article is to delineate the most frequent major complications that occur in patients with IE before, during, and after cardiac surgery, with particular emphasis on the incidence, pathophysiology, diagnostic, and treatment options.

## 2. Preoperative Complications

Besides the risk of death, IE is associated with a plethora of complications. Those complications can result from direct damage to the heart or surrounding structures, embolization from vegetations, or systemic hypoperfusion due to septic or cardiogenic shock. The incidence of overall complications is high: according to the literature, the incidence of at least one IE-related major complication varies between around 40% [12] and up to 70% in older studies [13].

When complications occur, the deterioration of patient status may necessitate ICU admission, e.g., when end-organ injury or heart failure is present [4]. While the exact percentage of patients with IE requiring ICU care prior to surgery is unknown [14], the reasons for ICU admission are primarily attributed to congestive heart failure (64%), septic shock (21%), neurological deterioration (15%), and cardiopulmonary resuscitation (9%). Once in the ICU, the vast majority of patients require inotropes or vasoconstrictors, and multiorgan failure occurs in up to two-thirds of the individuals [15]. Accordingly, mortality is high in those who are critically ill (40% in hospital [16] and 70% within 5 years [17]). Additionally, excessive mortality (95%) is seen in those patients who were assigned to ICU with indications for surgical interventions but deemed unfit for surgery [17].

Close monitoring is necessary to evaluate the effectiveness of the treatment, identify potential complications, and determine the need for surgery. Treatment decisions should be made by a multidisciplinary team on a case-by-case basis. As follows, we will delineate the most relevant IE-related complications that perioperative physicians face before surgery (see Table 1 for a synopsis).

### 2.1. Preoperative Cardiac Complications

Cardiac complications are the most common complications seen in IE patients, occurring in about 30–40% of cases [18], with a major impact on morbidity and mortality. Furthermore, cardiac complications are by far the most common indication for valve surgery [2,6].

#### 2.1.1. Heart Failure

The pathophysiology of heart failure (HF) in IE is multifactorial and can be attributed to several mechanisms. The infection of the endocardial structures, including heart valves and surrounding tissues, leads to various pathological processes. HF in IE is primarily a consequence of valvular dysfunction, with regurgitant flow leading to volume overload. Other, less frequent mechanisms are valve obstruction, intracardiac fistulas, myocarditis and sepsis, leading to myocardial inflammation and cardiac dysfunction [2].

HF is the most common cardiac complication in IE [2], with a prevalence between 30 and 50% in left-sided IE. Furthermore, HF is the single highest cause of death and also the most frequent indication for (urgent) cardiac surgery [5,6,19,20,21,22,23,24].

The diagnosis of HF in IE is primarily based on clinical presentation and confirmed with echocardiography [2,11], with additional imaging on indication [25]. Other diagnostic tools, such as electrocardiography (ECG), (invasive) hemodynamic monitoring, and cardiac biomarkers, may also be used to assess the extent of myocardial damage and circulatory dysfunction in the perioperative period [3].

The treatment of HF in IE requires a multidisciplinary approach and may include diuretics, inotropic support, and mechanical ventilation. Aggressive management of the underlying infection with appropriate antimicrobial therapy is also essential. Of note, cardiac surgery has been strongly associated with lower mortality when indicated [20,24], but is performed on only approximately half of patients with HF, mainly because of the surgical risk considered prohibitive to perform surgery [24]. Ideally, the decision, timing, and indication for urgent surgery due to HF should be taken within the endocarditis team [10].

#### 2.1.2. Cardiogenic Shock

When compensatory mechanisms fail in acute HF, cardiogenic shock (CS), the most severe form of HF, defined as a state of critical end-organ hypoperfusion due to reduced cardiac output [26], can occur. The abundance of CS in IE is strongly associated with adverse outcome, and in-hospital mortality exceeds 50% [23]. CS as a complication of IE has an estimated prevalence of 2–5% [6,12,23].

The diagnosis of CS in IE is based on clinical presentation and confirmed with invasive hemodynamic monitoring. Echocardiography is crucial to assess the extent of valvular dysfunction and myocardial damage. The management of CS in IE requires prompt recognition and aggressive intervention, including inotropic support, mechanical ventilation, and early surgical intervention [23], eventually as emergency or salvage surgery, to address the underlying valvular dysfunction.

#### 2.1.3. Conduction Abnormalities

The occurrence of conduction abnormalities, such as heart block or arrhythmias, typically indicates that the infection has extended beyond the valve annulus and infiltrated the surrounding tissue. Both the aortic and mitral valves are located close to the conduction system and atrioventricular node. Therefore, the spreading of the infection beyond the valve annulus and eventually the formation of a myocardial abscess can potentially affect these crucial conduction structures. The progression of infection through contiguous spread suggests a more advanced stage of the disease, which may explain the higher mortality observed in these individuals. The incidence of new-onset conduction abnormalities in IE has been shown to be around 5% in recent studies [6,12,27], while older studies found heart blocks in up to 14% of cases [28]. The most common conduction abnormality seen in IE is atrioventricular block [27].

The diagnosis of conduction abnormalities in IE is based on clinical presentation and confirmed with ECG. Further diagnostic tools, such as electrophysiological studies, may also be used to assess the extent of conduction defect. Management depends on the severity of the underlying conduction defect. Treatment strategies may include temporary pharmacological treatment, pacing, or permanent pacemaker implantation. However, patients with heart block are at increased risk of adverse outcome, and immediate surgical intervention should be considered [27].

#### 2.1.4. Intracardiac Abscess 

Intracardiac abscesses are caused by the spread of the bacterial infection from the endocardial surface of the heart, leading to the formation of a pocket of pus within the heart. Periannular extension of IE from the aortic valve, primarily to the aortic root, is the most common site of intracardiac abscess, followed by extension of the infection from the mitral valve. [29,30]. The incidence of intracardiac abscess varies in the literature from 12 to 35% [5,6,21,29,31]. Periannular infection can lead to the formation of intracardiac fistulas in 1–3% of cases [32].

Diagnosis of intracardiac abscess can be challenging due to the variable clinical presentation. Diagnostic modalities include transthoracic (TTE) or transesophageal echocardiography (TEE) and computed tomography (CT) or magnetic resonance imaging (MRI). The treatment of intracardiac abscesses in IE requires a combination of medical and surgical management. Antibiotic therapy is necessary to treat the underlying infection, and surgical intervention is indicated to eradicate infected tissue and restore cardiac structures, which may include aortic root replacement [33] or the reconstruction of the intervalvular fibrous body [34].

#### 2.1.5. Coronary Events

Coronary events are a rare but potentially life-threatening complication of IE that can result in acute coronary syndrome, myocardial infarction, or sudden cardiac death. They are primarily caused by coronary embolization or, less frequently, by abscess, pseudoaneurysm, or large vegetations [35]. Coronary events in IE occur in ca. 2–6% of patients, the incidence of heart failure is increased to 70%, and mortality is doubled [12,35,36].

Diagnosis requires a high degree of suspicion and can be challenging due to the variable clinical presentation. Diagnostic modalities may include ECG, TTE, CT, cardiac biomarkers, and coronary angiography. Due to the limited occurrence of coronary events in patients with IE, there is a lack of comprehensive and definitive recommendations in the existing literature regarding their management. Various treatment options have been suggested, including thrombolytic therapy, percutaneous revascularization with thrombectomy, balloon dilatation with or without coronary artery stent placing, surgical embolectomy, or (emergency) aortic valve replacement if coronary compression is caused by an aortic abscess.

### 2.2. Preoperative Noncardiac Complications

#### 2.2.1. Neurological Complications

Symptomatic neurological complications occur in 15 to 30% of patients, and silent events, as can be assessed by imaging such as MRI, are even more frequent [37,38]. Neurological complications represent the most common extracardiac complication of IE and can result in significant morbidity and mortality [39,40,41].

Following a neurological event, the need for cardiac surgery often remains or even becomes more urgent. The indication and especially timing of surgery are multidisciplinary decisions that must be carefully weighed against the risks (e.g., intracranial bleeding) during the perioperative period and the expected outcome after surgery. However, the risk of postoperative neurological decline is minimal after a silent cerebral emboli or transient ischemic attack, and surgery should proceed promptly if there is a continuing indication for a surgical intervention [2,42].

##### Ischemic Stroke

Septic embolism arising from vegetations is widely acknowledged as the primary mechanism attributed to the brain lesions observed in IE. However, other alternative pathomechanisms, such as cerebral small-vessel vasculitis, might also be involved [42]. The incidence of stroke in IE is generally reported to be 20–40% of cases, and risk factors for cerebral embolization are vegetation size and mobility, *S. aureus* infection, and mitral valve involvement [5,6,21,22,39,43,44].

The diagnosis of stroke in IE requires a high degree of suspicion and can sometimes be challenging due to the variable clinical presentation. Diagnostic modalities may include CT or the MRI of the brain. Treatment of stroke in IE may involve a combination of medical and interventional management. Antibiotic therapy is paramount to treating the underlying cardiac infection. The use of thrombolytic therapy is not recommended in this patient population due to the increased risk of hemorrhage [45], but interventional thrombectomy may be considered in selected patients [46]. Furthermore, when indicated, urgent valve surgery appears to be safe in most cases, even in the presence of minor ischemic stroke, and can even improve outcomes [41]. However, determining the optimal timing for surgery in patients with symptomatic ischemic stroke and a moderate-to-large infarcted territory presents additional challenges due to the risk of perioperative hemorrhagic transformation in the infarcted tissue, which is based on the exposure to high doses of anticoagulation during cardiopulmonary bypass. In such cases, it may be prudent to consider a waiting period of 2 to 4 weeks after the initial cerebrovascular event before proceeding with surgery [47].

##### Infectious Neurological Complications

Infectious neurological complications in IE can result from the direct invasion of the brain tissue by the infective organism or from an immune-mediated response. *Infective intracranial aneurysms* arise from the migration of septic emboli to the vasa vasorum of intraluminal spaces, followed by the contiguous spread of infection toward the vessel wall. The prevalence is estimated to be around 2 to 4% [40]. The incidence of IE-associated *meningitis* is similarly 1–5% [39,48], and *brain abscess* variably occurs in 1–7% of cases [39,41].

The diagnosis of infectious neurological complications in IE comprises non-invasive imaging (CT, CT angiography (CTA), MRI), cerebrospinal fluid analysis, and conventional neurological angiography in selected cases. Concerning treatment, adequate antibiotic therapy is pivotal to treating the underlying infection, and percutaneous or surgical interventions may be necessary to drain any abscesses or remove any infected tissue [42].

##### Intracranial Hemorrhage

Intracranial bleeding can present as primary intraparenchymal hemorrhage, e.g., as consequence of a ruptured mycotic aneurysm, cerebral microbleeds, or hemorrhagic conversion of an ischemic stroke. The underlying mechanism for the development of primary intracranial hemorrhage in IE is believed to be related to septic embolization into the cerebral vasculature. This can result in either outright vessel rupture or vessel incompetence [47,49]. The incidence of intracranial hemorrhage in IE is 4–11% [21,39,41,50]. In contrast, cerebral microbleeds are much more common, with a prevalence between 60 and 90% [40,51].

Diagnostic modalities include CT, CTA, conventional angiography, or MRI, and treatment again involves early adequate antimicrobial treatment, temporary discontinuation of anticoagulant therapy, and, in exceptional cases, neurosurgical interventions. In these patients with intracranial bleeding, although they have a per se increased risk of death [39], neurosurgery itself does not further increase long-term mortality [52]. Furthermore, patients with intracranial hemorrhage have a higher mortality when undergoing cardiac surgery, but additional neurological deterioration caused by surgery in case of survival is rare [50].

#### 2.2.2. Non-Stroke Embolization

Non-stroke embolization in IE occurs as a vegetation dislodges, fragments into smaller particles, and subsequently traverses the bloodstream. These particles can obstruct individual blood vessels in different areas, leading to a double-hit injury. This injury comprises an ischemic insult due to the blockage of blood flow and an inflammatory/infectious insult resulting from the presence of inflammatory and infectious components within the obstructed vessel.

The incidence of symptomatic non-stroke embolization in IE varies and is 20–50% [5,6,21,22,53,54,55]. Embolization can potentially affect any organ, but the most frequently involved organ is the spleen, followed by the kidneys, skin, and extremities, as well as the lungs in right-sided IE. Here, these emboli can lead to infarction and subsequent organ dysfunction [2].

Diagnosis depends on the affected organ and, alongside clinical examination, several imaging modalities, including ultrasound, CT, CTA, MRI, or angiography. Management varies depending on the location and severity of the embolic event. Beside symptomatic therapy, surgical embolectomy, splenectomy, percutaneous drainage of abscess, or endovascular interventions may be necessary in certain cases [56].

#### 2.2.3. Acute Kidney Injury

Acute kidney injury (AKI) in IE results from multiple factors, including immune-mediated glomerular injury, septicemia-induced hypotension, renal ischemia caused by septic emboli, or antibiotic/contrast agent-mediated nephrotoxicity [57]. The incidence is 6–30% of cases [2,6,12,21,58], depending on the study and patient population. The development of AKI in IE is associated with increased mortality and morbidity [2].

Diagnosis is based on the combination of laboratory parameters, clinical findings, and imaging studies according to available guidelines [59]. In selected patients suspected of having immune-complex-mediated renal injury related to IE, renal biopsy can be indicated. The latter may identify specific forms of immune-mediated glomerulonephritis that may be amenable to specific therapies such as corticosteroids. The treatment of AKI in IE includes the management of the underlying infection, the optimization of hemodynamics, and supportive care for renal function. Volume resuscitation, blood pressure support, and the avoidance of nephrotoxic medications are essential in managing AKI. In severe cases, renal replacement therapy may be necessary [2].

#### 2.2.4. Sepsis/Septic Shock

Sepsis in IE results from the spread of the infectious agent from the endocardial surface to the bloodstream. This leads to activation of the host immune system and the release of proinflammatory cytokines, resulting in a systemic inflammatory response. In severe cases, this can progress to septic shock, which is characterized by hypotension and potential multiorgan dysfunction.

The incidence of sepsis and septic shock in IE varies depending on patient-related factors and infective agents, but it is estimated to be 6–20% of cases. Patients with IE in septic shock have exceedingly poor outcomes [6,12,60,61,62].

The diagnosis of sepsis and septic shock in IE is generally based on clinical criteria as assessed by (advanced) hemodynamic and end organ monitoring [63]. Management requires a multidisciplinary approach in accordance with international guidelines [63] and includes the appropriate antimicrobial and supportive therapy for organ dysfunction, as well as, eventually, surgical interventions. However, surgical treatment has a positive effect on outcomes in these patients [64]. Given the complex nature of decision-making in this critically ill patient population, where indications and contraindications for cardiac surgery may coexist, it is crucial to approach these cases within a collaborative and multidisciplinary setting, such as the endocarditis team.

#### 2.2.5. Pulmonary Complications

Pulmonary complications in IE, predominantly in right-sided IE and pacemaker lead IE, can result from septic embolization, immune-mediated injury, and direct pulmonary infection. Septic emboli can cause pulmonary embolism, leading to pulmonary infarction, abscesses, and pleural empyema. Immune-mediated injury can result in vasculitis and diffuse alveolar hemorrhage, and direct infection of the lung can cause pneumonia [65]. The incidence of pulmonary complications in IE ranges from 4 to 10%, depending on the patient population. Patients with a cardiac implantable electronic device (CIED) and active intravenous (iv) drug abuse are particularly at risk for pulmonary complications [6,21,65].

Diagnostic tools for pulmonary complications in IE include chest X-rays and CT scans of the chest. These imaging studies can detect septic emboli, abscesses, and pleural effusions. In some cases, bronchoscopy may be necessary to diagnose pneumonia and rule out other causes of pulmonary infiltrates. The treatment of pulmonary complications in IE includes management of the underlying infection, antimicrobial therapy, and supportive care. The drainage of pleural effusions and abscesses may be necessary. In severe cases, surgical pulmonary intervention may be required [2].

## 3. Intraoperative Complications

Intraoperative complications and their prevention are an important aspect of the surgical management of IE. Treatment can be complex, with some specific issues relevant for cardiac surgeons, anesthesiologists, and perfusionists [3]. Compared to medically treated patients, however, the scientific evidence on the intraoperative period is utterly sparse. In this section, we will discuss the pathophysiology, incidence, diagnosis, and treatment of intraoperative complications in patients with IE, with a focus on hemodynamic instability, coagulopathy, and bleeding (see also Table 2).

### 3.1. Hemodynamic Instability 

Hemodynamic instability during surgery for IE can result from various factors, including the patient’s underlying cardiac function, volume status, and systemic inflammatory response. Pre-bypass, up to 40% of all patients suffer from HF, due to either preexistent cardiac dysfunction and/or volume overload due to valve regurgitation [2]. Additionally, patients with sepsis can present with systemic vasoplegia and septic cardiomyopathy, the latter being defined as a sepsis-associated acute syndrome of non-ischemic cardiac left and/or right ventricular systolic and/or diastolic dysfunction [66]. 

With long cross-clamp and cardiopulmonary bypass (CPB) time in extensive cardiac surgery, the risk of post-bypass low cardiac output syndrome (LCOS) may increase. Evidence suggests that patients with IE are more susceptible to cross-clamp and CPB time than other patients undergoing cardiac surgery [3,67]. 

The exact incidence of LCOS, difficulty or failure to wean from CPB, or vasoplegia during surgery for IE is unknown. Moreover, it is currently not established whether the risk of hemodynamic instability is indeed higher in patients with IE compared to patients undergoing cardiac surgery for other indications [3].

(Extended) hemodynamic monitoring, including invasive blood pressure monitoring and the measurement of cardiac output or systemic vascular resistance, may be indicated to detect and manage hemodynamic instability. Intraoperative TEE is also inevitable to evaluate ventricular or valvular dysfunction. Furthermore, the direct or indirect assessment of potential end-organ ischemia by monitoring tissue oxygen saturation using cerebral oximetry and monitoring urine output or lactate levels in hemodynamically unstable patients should be considered [68].

The treatment of hemodynamic instability may include aggressive fluid resuscitation, vasopressor support, and inotropic agents. Pre-bypass, hemodynamic goals depend largely on IE-related cardiac pathologies, which are mostly aortic and/or mitral valve regurgitation [3,69]. There is almost no literature on hemodynamic management, specifically in patients with vasoplegia; the sometimes-promoted prophylactic application of methylene blue does not appear to be beneficial concerning vasopressor requirements [70]. Furthermore, intraoperative hemo-adsorption of cytokines during bypass did not show any clinically meaningful advantage on hemodynamics in patients with IE [71].

Hence, until further evidence exists, hemodynamic management during cardiac surgery for IE will need to rely on general pathophysiological considerations, institutional algorithms, and expert opinions or recommendations [72,73,74].

### 3.2. Coagulopathy and Bleeding

IE is associated with a substantial and intricate interplay between inflammation and coagulation, a phenomenon that has been referred to as “immunothrombosis” [75]. This interaction has a significant role in the pathogenesis of IE, as various steps in the coagulation cascade are influenced by this reaction [76]. Generally, hypercoagulation is present in IE [77,78]. However, during cardiac surgery for IE, the occurrence of severe coagulopathy and bleeding is common. Intraoperative coagulopathy and bleeding in IE can result from various factors, including the patient’s underlying coagulation status, the use of anticoagulant agents, surgical trauma, and infection-related factors such as platelet dysfunction and disseminated intravascular coagulation (DIC) [79,80]. Furthermore, many antibiotics that are used to treat IE can affect coagulation, either by direct interaction with the coagulation system or via drug–drug interaction, such as, e.g., between cefazoline and vitamin k antagonists [3].

The precise incidence of coagulopathy and excessive bleeding during surgery for IE is not known, but the percentage of patients receiving the transfusion of blood products has been reported to be as high as 80% [81]. Also, patients with IE are overrepresented in cohorts requiring massive transfusion [82,83]. Furthermore, it is not entirely clear whether cardiac surgery for IE is per se associated with an increased risk of bleeding. While the transfusion rate is indeed higher in IE [79], after correction for risk factors such as age, sex, BMI, or anemia, IE was not independently associated with blood transfusion [84]. The incidence of DIC is between 1and 20%, and the occurrence of DIC is associated with increased mortality [12,85].

The diagnosis of deranged coagulation during surgery for IE may be challenging due to the need for rapid decision-making and intervention. Hemostatic monitoring, including laboratory coagulation studies and point-of-care testing (POCT) of coagulation, is essential for detecting and managing coagulopathy and bleeding. Notably, coagulopathy in IE can, at least in part, be assessed using POCT, such as thromboelastography (TEG) [78] and rotational thromboelastometry (ROTEM) [79].

The management of coagulopathy and bleeding in IE includes transfusion of allogenic blood products, including packed red blood cells, fresh frozen plasma, and platelets. The application of coagulation factors, ideally based on POCT-guided algorithms, is also common [86]. The use of acute normovolemic hemodilution is not the standard of care and is controversially discussed [87,88]. In contrast, the use of intraoperative cell salvage is very common in cardiac surgery, although in systemic infection, such as IE, the fear of exacerbation of inflammatory response may preclude its use. The limited available evidence, however, shows no deleterious effect of cell salvage and re-transfusion during cardiac surgery for IE [89]. Future research will need to further unravel the exact mechanisms of perioperative coagulopathy in IE to improve coagulation management during and after surgery.

## 4. Postoperative Complications

Postoperative complications, usually present within the first 24 h after surgery [14], can arise from various sources, including the patient’s underlying medical conditions, the surgical procedure itself, and infection-related factors such as the systemic inflammatory response. Below, we will delineate pathophysiology, incidence, diagnostic possibilities, and management of the most relevant complications that need to be considered directly after surgery on the ICU, but also in the long-term follow-up of the respective patients (see also Table 3).

### 4.1. Vasoplegic Syndrome

The pathophysiology of vasoplegia in IE is not fully understood but is believed to be associated with an exaggerated inflammatory response and the release of endothelial nitric oxide triggered by sepsis and heart failure. These factors are further amplified during CPB, contributing to the development and worsening of vasoplegic syndrome [90]. The incidence of postoperative vasoplegia in patients with IE has been reported to be about 30% [91]. The percentage of patients with the postoperative need for inotropes and/or vasopressors after surgery for IE can be as high as 90% [92].

The diagnosis of vasoplegic syndrome in IE is based on clinical presentation and echocardiography to rule out cardiac dysfunction, (extended) hemodynamic monitoring, and laboratory parameters. Although there is no universally accepted definition, patients with vasoplegic syndrome typically present with severe hypotension and low systemic vascular resistance, despite adequate fluid resuscitation and high-dose vasopressor support [93].

Management strategies in postoperative vasoplegic syndrome include, beside adequate fluid resuscitation, high-dose vasopressor support, including standard catecholamine agents such as norepinephrine, epinephrine, and phenylephrine, as well as alternative pharmacological agents such as vasopressin, methylene blue, hydroxocobalamin, angiotensin II, and corticosteroids [73,93]. In severe cases, extracorporeal membrane oxygenation (ECMO) may be necessary to maintain end-organ perfusion. In this respect, there is currently no evidence for a specific approach in patients with IE compared to other cardiac surgery [3]. Intraoperative hemo-adsorption does not impact postoperative vasopressor requirements in IE [71].

### 4.2. Acute Kidney Injury

In addition to the pathophysiology of pre-existing AKI, postoperative new-onset or the progression of AKI and renal failure is likely to be multifactorial and results from ischemic injury, inflammation, and oxidative stress. Cardiopulmonary bypass, hypotension, and exposure to nephrotoxic agents can also contribute to postoperative renal injury. The incidence of postoperative AKI in patients with IE varies widely, ranging from 30 to 60%, depending on the study and patient population [55,94,95], while renal failure, requiring renal replacement therapy, occurs in 6–20% of patients [55,96,97,98].

The diagnosis and treatment of postoperative AKI and renal failure in IE, predominantly in the ICU setting, does not significantly differ from the management of AKI before surgery and involves a combination of supportive care, optimizing hemodynamics, avoiding nephrotoxic agents while continuing adequate antimicrobial therapy, and maintaining adequate hydration. In acute (or chronic) renal failure, renal replacement therapy in hemodynamically unstable patients would likely rather be treated with continuous venovenous hemofiltration [4].

### 4.3. Sepsis

Postoperative sepsis, either preexistent or developing during or after surgery, is potentially aggravated by the impact of cardiac surgery, ischemia/reperfusion, and CPB and can lead to systemic inflammatory response syndrome and life-threatening multiple organ dysfunction. Circulating proinflammatory cytokines, as well as the liberation of infective material during surgical removal of infected tissues, may contribute to postoperative sepsis and septic shock [99,100].

The incidence of postoperative sepsis in patients with IE is 13–22% [55,95,96]. The presence of hypotension and decreased systemic vascular resistance, despite adequate fluid resuscitation and the administration of adrenergic vasopressors, while maintaining high cardiac output is characteristic of postoperative sepsis.

Treatment of postoperative sepsis in IE should follow the general guidelines of sepsis treatment, including supportive care, appropriate antibiotic therapy, fluid resuscitation, and vasopressor treatment [63]. A more pathophysiology-oriented approach is to scavenge circulating proinflammatory cytokines by incorporating hemo-adsorption filters into the cardiopulmonary bypass system [101]. However, despite several smaller, mainly retrospective studies showing reduced incidence and sepsis-related mortality in patients with IE [102,103,104], there is up to now only one randomized controlled trial published showing that, while plasma cytokines were reduced at the end of CPB with hemo-adsorption, no significant differences were observed in any of the clinically relevant outcome measures [71]. At this point in time, hemo-adsorption cannot be advocated for all cardiac surgeries in patients with IE.

### 4.4. Postoperative Stroke

Postoperative stroke in IE can be caused by the aggravation of preoperatively abundant (silent) stroke or can be a result of a new embolic event due to vegetations, air, or calcified plaques, potentially in concert with hypoperfusion during surgery.

The incidence of postoperative stroke is approximately 2–11% [55,97,98]. Early clinical examination after emergence from anesthesia is crucial to detect neurological deficits. Imaging studies such as CT or MRI can help to confirm the diagnosis of stroke and determine the location and extent of brain injury.

Treatment options are limited in many cases. In selected patients, intra-arterial thrombolysis and/or endovascular mechanical thrombectomy might be performed after carefully weighing the risks and benefits [105].

### 4.5. Re-Exploration Due to Bleeding

Surgical re-exploration due to bleeding may be primary surgical of origin, a consequence of ongoing coagulopathy, or the combination of both. The incidence of re-exploration is reported at 8–12% [55,95,96,97] compared to 2–8% in general cardiac surgery [106].

Increased chest tube drainage or signs of cardiac tamponade, which should be quickly confirmed via echocardiography, accompanied by laboratory parameters and the hemodynamic situation, will form the basis to indicate surgical re-exploration. The correction of coagulation and the transfusion of blood products, ideally guided by POCT-driven algorithms, should be implemented. In the case of ongoing bleeding or tamponade, repeat sternotomy or subxyphoidal evacuation of blood may be necessary. Thorough and precise surgical technique, along with systematic intraoperative inspection of potential sites of bleeding, including the implementation of hemostasis checklists [107], can help minimize the risk of encountering this complication [106].

### 4.6. Respiratory Insufficiency

Postoperative respiratory insufficiency in patients undergoing cardiac surgery for IE can result from multiple underlying pathophysiological factors, some of which may have been already present before surgery, including the effects of CPB and reperfusion injury, inflammatory response, fluid shifts, atelectasis, and deteriorated ventricular function after surgery, which may all contribute to the development of pulmonary edema, leading finally to respiratory compromise.

The incidence of postoperative respiratory insufficiency varies (6–24% of cases [55,96,97,108]) and depends on multiple factors, including patient characteristics, the extent and location of cardiac involvement, and the presence of pre-existing lung conditions, and is reported in.

Diagnosing postoperative respiratory insufficiency involves careful clinical evaluation and monitoring of respiratory parameters, chest X-ray, and CT. Treatment is a combination of supportive measures and targeted interventions, such as ensuring adequate mechanical ventilation with appropriate ventilator settings, careful fluid administration, early mobilization, and chest physiotherapy [109]. The prophylactic or therapeutic perioperative use of inhaled pulmonary vasodilators might benefit in selected cases [110], while more evidence is needed to support their use.

### 4.7. Permanent Pacemaker Requirement

The initial infection itself, inflammation and fibrosis, or cardiac surgery can cause disturbances in the conduction system. In the case of irreversible damage to the conduction pathways, permanent pacemaker dependency may occur. The type and extent of cardiac surgery and the presence of pre-existing conduction abnormalities determine the incidence, ranging from 2 to 13% of patients requiring permanent pacemaker implantation following cardiac surgery for IE [55,97,111,112].

Continuous ECG monitoring can detect conduction abnormalities or persistent arrhythmias. The management of postoperative permanent pacemaker requirements involves collaboration between cardiac surgeons, cardiologists, and electrophysiologists. Key aspects of management include temporary epicardial or transvenous pacing, pacemaker selection (single-chamber or dual-chamber), the optimal timing of placing the permanent device in an infection-free interval, and regular follow-up and monitoring to assess pacemaker function and address any issues or complications [111].

## 5. Conclusions

This narrative provides an overview of perioperative complications in IE that all healthcare providers who are involved in the management of surgically treated patients with IE should be aware of.

The current evidence suggests that a multidisciplinary approach involving cardiologists, infectious disease specialists, cardiac surgeons, and other healthcare professionals such as cardiac anesthetists is crucial for the optimal management of IE and its associated complications.

Furthermore, patients who survived an episode of IE carry a life-long risk of long-term complications, such as IE recurrence, arrhythmias, stroke, myocardial infarction, heart failure, and sudden cardiac death [98,112].

While significant progress has been made in the understanding and management of perioperative complications in IE, there are still several areas that require further research and exploration. Future directions should include prospective studies to determine optimal strategies for risk stratification, diagnostic algorithms, and treatment interventions. Additionally, long-term follow-up studies are needed to assess the impact of perioperative complications on patient outcomes and quality of life.

## Figures and Tables

**Table 1:** **Preoperative Complications:** Synopsis of the most relevant complications, including frequency, diagnostic, and treatment options that patients with IE may suffer from before cardiac surgery is performed. TTE, transthoracic echocardiography; TEE, transesophageal echocardiography, ECG, electrocardiogram; CT, computed tomography; CTA, CT angiography; MRI, magnetic resonance imaging; PET-CT, Positron emission tomography–computed tomography; PCI, percutaneous coronary intervention.

Complication	Frequency	Diagnostic Tools	Management
**Cardiac**			
Heart Failure Cardiogenic shock	30–50% 2–5%	TTE, TEE, (extended) hemodynamic monitoring	Inotropes, mechanical support, (urgent/emergency) cardiac surgery
Conduction abnormalities	5–14%	ECG	Pacemaker, cardiac surgery
Intracardiac abscess	12–35%	TTE, TEE, CT, MRI, PET-CT	Cardiac surgery
Coronary events	2–6%	ECG, CT, coronary angiography	Cardiac surgery, PCI
**Noncardiac**			
Ischemic stroke	20–40%	CT, MRI	Symptomatic, thrombectomy
Infectious neurological complications	2–7%	CT, CTA, MRI, angiography	Symptomatic, neurosurgery, percutaneous intervention
Intracranial hemorrhage	4–11%	CT, CTA, MRI, angiography	Symptomatic, neurosurgery, percutaneous intervention
Non-stroke emboli	20–50%	Ultrasound, CT, CTA, MRI, angiography	Symptomatic, percutaneous or surgical embolectomy
Acute kidney injury	6–30%	Lab, urine output, imaging	Symptomatic, renal replacement therapy
Sepsis/septic shock	6–20%	(Extended) hemodynamic monitoring	Antibiotics, fluid and vasoactive agents
Pulmonary complications	4–10%	X-Ray, CT, ultrasound	Antibiotics, percutaneous or surgical intervention

**Table 2:** Intraoperative complications: common intraoperative complications, including frequency, diagnostic, and treatment options that patients with IE may suffer from during cardiac surgery. LCOS, low cardiac output syndrome, TEE, transesophageal echocardiography; POCT, point-of-care-testing.

Complication	Frequency	Diagnostic Tools	Management
Hemodynamic instability	unknown	TEE, (extended) hemodynamic monitoring	Inotropes, vasoactive agents, mechanical support
Vasoplegia			
LCOS			
Failure to wean			
Coagulopathy/Bleeding	unknown	Coagulation lab, POCT coagulation tests	Transfusion of blood products, coagulation factors
Thrombo-embolic			
Massive transfusion			

**Table 3:** **Postoperative complications.** Overview of the most relevant postoperative complications, including frequency and diagnostic and treatment options that patients with IE may suffer from after cardiac surgery. POCT, point-of-care-testing; TTE, transthoracic echocardiography; TEE, transesophageal echocardiography; ECG, electrocardiogram; CT, computed tomography; MRI, magnetic resonance imaging.

Complication	Frequency	Diagnostic Tools	Management
Vasoplegic syndrome	30%	TEE, TTE, (extended)hemodynamic monitoring	Vasoactive agents, mechanical support
Acute kidney injury	30–60%	Lab, urine output, imaging	Optimizing hemodynamics and hydration, avoiding nephrotoxic agents, renal replacement therapy
Sepsis/septic shock	13–22%	(Extended) hemodynamic monitoring	Antibiotics, fluid, vasoactive agents
Postoperative stroke	2–11%	CT, MRI	Symptomatic, thrombectomy, intra-arterial thrombolysis
Re-exploration due to bleeding	8–12%	Chest tube drainage, TTE, TEE, coagulation lab, POCT coagulation tests	Transfusion of blood products, coagulation factors, re-sternotomy
Respiratory insufficiency	6–24%	chest X-ray, CT	Symptomatic, (re-)intubation, mechanical ventilation
Permanent pacemaker requirement	2–13%	ECG	Temporary epicardial/transvenous pacing, permanent pacemaker

## Data Availability

Not applicable.
